# Mammalian Niche Conservation through Deep Time

**DOI:** 10.1371/journal.pone.0035624

**Published:** 2012-04-23

**Authors:** Larisa R. G. DeSantis, Rachel A. Beavins Tracy, Cassandra S. Koontz, John C. Roseberry, Matthew C. Velasco

**Affiliations:** 1 Department of Earth and Environmental Sciences, Vanderbilt University, Nashville, Tennessee, United States of America; 2 Department of Anthropology, Vanderbilt University, Nashville, Tennessee, United States of America; University of Copenhagen, Denmark

## Abstract

Climate change alters species distributions, causing plants and animals to move north or to higher elevations with current warming. Bioclimatic models predict species distributions based on extant realized niches and assume niche conservation. Here, we evaluate if proxies for niches (i.e., range areas) are conserved at the family level through deep time, from the Eocene to the Pleistocene. We analyze the occurrence of all mammalian families in the continental USA, calculating range area, percent range area occupied, range area rank, and range polygon centroids during each epoch. Percent range area occupied significantly increases from the Oligocene to the Miocene and again from the Pliocene to the Pleistocene; however, mammalian families maintain statistical concordance between rank orders across time. Families with greater taxonomic diversity occupy a greater percent of available range area during each epoch and net changes in taxonomic diversity are significantly positively related to changes in percent range area occupied from the Eocene to the Pleistocene. Furthermore, gains and losses in generic and species diversity are remarkably consistent with ∼2.3 species gained per generic increase. Centroids demonstrate southeastern shifts from the Eocene through the Pleistocene that may correspond to major environmental events and/or climate changes during the Cenozoic. These results demonstrate range conservation at the family level and support the idea that niche conservation at higher taxonomic levels operates over deep time and may be controlled by life history traits. Furthermore, families containing megafauna and/or terminal Pleistocene extinction victims do not incur significantly greater declines in range area rank than families containing only smaller taxa and/or only survivors, from the Pliocene to Pleistocene. Collectively, these data evince the resilience of families to climate and/or environmental change in deep time, the absence of terminal Pleistocene “extinction prone” families, and provide valuable insights to understanding mammalian responses to current climate change.

## Introduction

Understanding mammalian responses to climate change both today and in the past is critical to predicting potential responses to future climate change. Currently, mammals change their distributions, abundances, and phenology in response to ongoing climate change (e.g., [1]–[4]). Mammals have also demonstrated dramatic dietary responses to interglacial warming during the Pleistocene [5]. This dietary plasticity documents the adaptability of mammals to change their dietary behavior in response to available resources and falsifies the idea that dietary niches are conserved over time. However, there is also evidence that the niches of mammals based on temperature and precipitation are conserved during the last glacial to interglacial transition [6]. Similarly, the conservation of niches, as determined by relative range size, occurs above the species level during the late Pleistocene to late Holocene [7]. As the majority of bioclimatic envelope models predict future species distributions based on the assumption that niches are conserved over time, it is critical to further test if niche conservation occurs at higher taxonomic levels through deep time.

Niche conservatism can be defined as the capacity of a species to retain components of their fundamental niche over time [8] or the phenomenon that enables species to persist in ecological environments over time [7]. Thus, niche conservatism promotes the maintenance of species distributions over time; however, controls on niche conservatism vary from the species to higher taxonomic levels [7]. Recent reviews on the prevalence of niche conservatism reveal that ‘niche conservatism’ is generally widespread, although it is often defined and assessed using diverse methods (e.g., [8]–[10]). In many cases range area is used as a proxy for an organism's niche, and compared through time (e.g., [6], [7]). Although range area is likely reflecting an organism's realized niche, it is often the best proxy available for assessing ecological and/or climatic niches as species ranges encompass habitats, dietary resources, and thermal conditions that allow for their survival. Range areas can also be assessed at multiple taxonomic scales (e.g., species to family) today and compared through time via historic and fossil records. For example, Hadly et al. [7] found that at the genus and family levels, range sizes were relatively consistent between the late Pleistocene and late Holocene, suggesting that niche conservatism occurs above the species level.Conversely, species within a genus may divide up niche space based on the availability of resources and subsequently be more susceptible to fluctuating climates and environmental resources [7].

While much debate has focused on whether or not niches are conserved, there is a need to better understand the temporal dimensions of ecological niche conservation [10]. Furthermore, Wiens and Graham [8] suggest focusing on the effects of niche conservatism. For example, does niche conservatism affect a taxon's vulnerability to extinction? This perspective may clarify potential causal factors behind the terminal Pleistocene extinction. As human activities have directly and indirectly impacted biodiversity through time and space via habitat fragmentation, hunting/fishing, the introduction of invasive species, global warming, and the synergistic effects of multiple factors [11]–[15], many [16]–[19] argue that human activity, rather than climate change, was the primary cause of Pleistocene megafaunal extinctions. In order to elucidate the variety of causal factors (human and non-human) which may have contributed to species extinction during the Pleistocene, we must first understand how mammals altered their niches through time and if families that went locally extinct in North America were already declining in range size. Similar work examining range shifts during the late Pleistocene demonstrates that Pleistocene survivors and victims responded similarly [20]; however, little is known about how families alter their niches over deep time including prior to the Pleistocene.

Through a meta-analysis of North American mammalian range changes from the Eocene through the Pleistocene, we build on previous work [7], [20]–[21] to determine whether relative range size is conserved at the family level for North American mammals, including those that did not survive the terminal Pleistocene extinction. We add to the families studied by Hadly et al. [7], in the contiguous United States, and extend their work in deep time to include epoch-scale time bins for the Eocene through the Pleistocene. Herein, we use the mammalian fossil record (via the Paleobiology Database [22]) to determine absolute and relative range sizes (a proxy for an organism's realized ecological niche) that allow us to ask the following principal questions: (*i*) are mammalian ranges conserved at higher taxonomic levels through deep time, (*ii*) do environmental and/or climatic changes affect relative and/or absolute range sizes differently at higher taxonomic levels, and (*iii*) how does taxonomic diversity within a family (e.g., the number of genera and/or species) affect relative range size? Furthermore, we quantify changes in the centroids of range area polygons over time to assess if centroid locations shifted south through time, possibly in response to post-Eocene cooling. The analysis of mammalian range changes through deep time can clarify how climate and environmental changes affect mammalian families and the potential influence of these variables on range conservation.

We also examine if particular niche characteristics influence range changes at the family level from the Pliocene to the Pleistocene. Specifically, we ask the following: (*i*) were families that went locally extinct in North America during the Pleistocene already on the decline, (*ii*) did maximum body size of family members affect changes in range size, and (*iii*) were particular orders and/or functional groups (e.g., ungulates, carnivorans) more successful at increasing their ranges since the Pliocene? If body size or other intrinsic life history characteristics are more susceptible to climate change, we expect that families containing megafauna would respond similarly. Likewise, higher level categorizations such as taxonomic orders or functional groups may also reflect similar responses to climatic changes.

Similar to previous studies that focused on the Pleistocene and Holocene [7], [20]–[21] we examined absolute range size (additionally standardizing ranges by using percent range area occupied, see Methods) and centroids of mammalian taxa; however, we also analyzed relative range sizes in accordance with Hadly et al. [7] to control for sampling biases (including taphonomy and varying continental land areas) across epochs. Furthermore, to examine range conservatism over deep time it was necessary to both examine taxa at the family level and compare across epochs. Although not all family groupings are monophyletic, mammals present less disagreement than other animals and plants, reflect evolutionary relationships, and share similar life history traits (e.g., equids, camelids, felids). Additionally, families persist over deep time (in contrast to genus and species groups) and fossils can typically be identified to both the family level and attributed to a given epoch.

## Results

### Geographic Range Size and Taxonomic Diversity

In order to assess overall trends in range expansion and contraction, we analyzed the absolute change in percent range area occupied from each epoch to the next consecutive epoch (see Methods; Table 1, S1, S2). There is no significant increase in percent range area occupied from the Eocene to the Oligocene (55% increased, *n* = 11, average change of +5.9%; Table S2). In contrast, from the Oligocene to the Miocene there was a significant increase in percent range area occupied (*n* = 18, average change of +31.6%; *p*<0.001 for all families and those with 10 or more localities, and *p* = 0.027 for families with 25 or more localities); 13 families increased while only 3 (Aplodontidae, Geomyidae, and Leporidae) decreased (net loss of 16.4, 7.4, and 2.3 percent range area occupied, respectively). Conversely, from the Miocene to Pliocene, there is no clear pattern with 13 of 25 families declining in percent range area occupied (average change of −4.0; Table S2). Of the families spanning the Pliocene to Pleistocene transition (*n* = 28), all except Antilocapridae (net loss of 6.2 percent range area occupied) increased their ranges (average change of +19.3%; *p*<0.0001 for all comparisons, regardless of number of localities, Table S2).

**Table 1:** Range size and rank of North American families from the Eocene to Pleistocene.

		Eocene			Oligocene			Miocene			Pliocene			Pleistocene		
Order	Family	Area (log10)	Area (%)	Rank (1–10)	Area (log10)	Area (%)	Rank (1–16)	Area (log10)	Area (%)	Rank (1–22)	Area (log10)	Area (%)	Rank (1–28)	Area (log10)	Area (%)	Rank (1–28)
Artiodactyla	*Antilocapridae* 1 *^,^* 3							6.57269	54.1	14	6.51652	58.5	13	6.56903	52.3	22
	*Bovidae* 1 *^,^* 3							5.74172	8					6.77614	84.2	
	*Camelidae* 1 *^,^* 2 *^,^* 3	6.23075	67.4	3	6.54893	58.4	4	6.74939	81.3	3	6.59166	69.5	3	6.69996	70.6	20
	*Cervidae* 1 *^,^* 3										6.53246	60.6	12	6.80297	89.5	4
	*Tayassuidae* 1 *^,^* 3				5.95903	15	10	6.74932	81.3	4	6.56579	65.5	8	6.73971	77.4	13
Carnivora	*Canidae* 1 *^,^* 3	5.55965	14.4	5	6.5869	63.8	2	6.7685	84.9	2	6.5785	67.4	6	6.74782	78.9	11
	*Felidae* 1 *^,^* 3							6.60684	58.5	11	6.6112	72.7	2	6.77551	84.1	7
	*Mustelidae* 1 *^,^* 3				6.23027	28.1	7	6.633	62.2	8	6.5559	64	10	6.79525	88	6
	*Procyonidae*							6.48085	43.8	19	6.40623	45.3	20	6.62359	59.2	21
	*Ursidae* 1 *^,^* 3	5.22216	6.6	8	3.00596	0.02	16	6.57436	54.3	13	6.46449	51.9	18	6.75037	79.3	9
Didelphimorphia	*Didelphidae*													6.0589	16.1	
Lagomorpha	*Leporidae* 1	6.00753	40.3	4	6.58522	63.5	3	6.62679	61.3	9	6.5121	57.9	15	6.75008	79.3	10
	*Ochotonidae*				5.39199	4.1		6.45613	41.4					5.82898	9.5	
	*Soricidae*	5.36707	9.2	7	5.51352	5.4	12	6.50592	46.4	16	6.49471	55.6	16	6.71709	73.5	18
	*Talpidae*				4.68682	0.8	14	6.4906	44.8	17	6.51488	58.2	14	6.70118	70.8	19
Perissodactyla	*Equidae* 1 *^,^* 2 *^,^* 3	6.35212	89.1	1	6.72548	87.8	1	6.8081	93	1	6.6633	82	1	6.80914	90.8	3
	*Tapiridae* 1 *^,^* 3	6.28185	75.8	2	5.4612	4.8	13	6.69213	71.2	7	5.92022	14.8	23	6.72212	74.3	17
Proboscidea	*Elephantidae* 1 *^,^* 2 *^,^* 3										6.12524	23.7	22	6.80056	89	5
	*Mammutidae* 1 *^,^* 2 *^,^* 3							6.54005	50.2	15	6.58386	68.3	4	6.8157	92.2	1
Rodentia	*Aplodontidae*	5.22061	6.6		6.13494	22.5		5.62366	6.1							
	*Castoridae* 1 *^,^* 3	4.44283	1.1	9	6.30807	33.6	6	6.71165	74.5	5	6.49313	55.4	17	6.72342	74.6	16
	*Cricetidae*	5.54788	14	6	6.18347	25.2	8	6.58155	55.2	12	6.57771	67.3	7	6.81069	91.2	2
	*Erethizontidae*													6.68693	68.5	
	*Geomyidae*				6.41874	43.3	5	6.39432	35.9	20	6.55928	64.5	9	6.72759	75.3	15
	*Heteromyidae*				5.80909	10.6	11	6.69604	71.9	6	6.22417	29.8	21	6.33841	30.7	24
	*Hydrochoeridae* 1 *^,^* 2 *^,^* 3										5.49689	5.6	25	6.00185	14.2	28
	*Sciuridae*	3.97993	0.4	10	6.17153	24.5	9	6.61973	60.3	10	6.53307	60.7	11	6.73726	77	14
	*Zapodidae*				4.21956	0.3	15	6.37896	34.6	21	4.72063	0.9	26	6.44037	38.9	23
Xenarthra	*Dasypodidae* 3										3.79121	0.1	28	6.19099	21.9	25
	*Glyptodontidae* 1 *^,^* 2 *^,^* 3										5.87638	13.4	24	6.0207	14.8	27
	*Megalonychidae* 1 *^,^* 3							6.48351	44.1	18	6.58004	67.7	5	6.74627	78.6	12
	*Megatheriidae* 1 *^,^* 2 *^,^* 3													4.86456	1	
	*Mylodontidae* 1 *^,^* 2 *^,^* 3							5.72828	7.7	22	6.45108	50.3	19	6.77072	83.1	8
	*Nothrotheriidae* 1 *^,^* 2 *^,^* 3													6.2761	26.6	
	*Pampatheriidae* 1 *^,^* 2 *^,^* 3										4.1234	0.2	27	6.18423	21.5	26
**Total Range Size**		**6.40229**			**6.78219**			**6.83943**			**6.74965**			**6.85093**		

Range sizes are converted to a log_10_ scale and a percentage based on the total range size of all terrestrial mammalian families included during each epoch. Rank of family within each epoch is relative to other families present in that epoch through the Pleistocene: Eocene-Pleistocene (*n* = 10), Oligocene-Pleistocene (*n* = 16), Miocene-Pleistocene (*n* = 22), Pliocene-Pleistocene (*n* = 28).

1 Families containing taxa that went extinct during the terminal Pleistocene extinction in North America [37].

2 Families completely absent in North America, excluding re-introductions.

3 Families containing at least one extinct or extant megafaunal taxon (≥45 kg; based on data from [22], [33], [39]–[42]).

During the Pleistocene, latitudinal and longitudinal extents are strongly correlated with log range size (R^2^ = 0.84, *p*<0.0001; R^2^ = 0.92, *p*<0.0001; respectively). Latitudinal extent and longitudinal extent independently account for 70% and 84% of the variance in range size (R^2^ = 0.70, *p*<0.0001; R^2^ = 0.84, *p*<0.0001). Together, latitudinal and longitudinal extent account for 90% of the variance in range size (R^2^ = 0.90, *p*<0.0001). The lowest maximum latitudinal and longitudinal extents (decimal degrees) occur during the Eocene (17.4 and 17.8, respectively), while the greatest latitudinal and longitudinal extents occur during the Pleistocene and Miocene (22.3 and 51.7, respectively), all demonstrated by the family Equidae. Some minor discrepancies exist between family range size and latitudinal/longitudinal extent. For example, Camelidae had the largest latitudinal extent in the Pleistocene (tied with Equidae), but was only ranked 21 in range size. Similarly, Tapiridae had the largest longitudinal extent in this epoch but fell below the median range size. However, as latitudinal and longitudinal extents of mammalian families are highly correlated with range size, we do not comprehensively discuss longitudinal and latitudinal extents of mammalian families as these metrics similarly quantify the same underlying patterns of family distributions.

**Figure 1:**
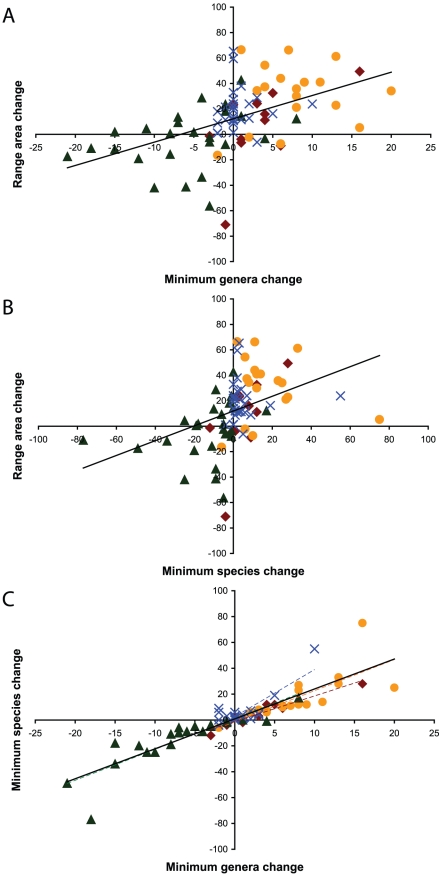
Relationships between changes in species, genera, and percent range area occupied through time. A) Minimum genera change and range area change (% occupied; R^2^ = 0.25, *p*<0.0001); B) minimum species change and range area change (% occupied; R^2^ = 0.17, *p*<0.001); and, C) minimum genera change and minimum species change (R^2^ = 0.79, *p*<0.0001) from the Eocene to Oligocene (red diamonds), Oligocene to Miocene (orange circles), Miocene to Pliocene (green triangles), and Pliocene to Pleistocene (blue Xs), with linear regression trend lines noted in solid black for all data and dashed colored lines corresponding to specific epochs (Eocene to Oligocene, R^2^ = 0.91, *p*<0.0001; Oligocene to Miocene, R^2^ = 0.56, *p*<0.001; Miocene to Pliocene, R^2^ = 0.76, *p*<0.0001; Pliocene to Pleistocene, R^2^ = 0.69, *p*<0.0001).

At any given epoch, there is a significant positive relationship between generic diversity (minimum number of genera per family, see Methods) and percent range area occupied (Table 2). The same relationship is true at the species level during each epoch, with the exception of the Pliocene where the relationship approaches significance (*p* = 0.057). Furthermore, the strength of these relationships are greatest during the Eocene-Miocene (R^2^ = 0.42 to 0.73), in contrast to the Pliocene and Pleistocene (R^2^<0.20 during the Pliocene and Pleistocene; Table 2). Similarly, taxonomic diversity (e.g., a net increase in minimum number of genera or species) is significantly positively related to net changes in percent range area occupied, when including all changes between consecutive epochs of all families from the Eocene to the Pleistocene (Figure 1, Table S3). However, changes between any two consecutive epochs in taxonomic diversity and percent range area occupied are not significantly related, with the exception of changes in net species diversity during the Eocene to the Oligocene (*p* = 0.037, R^2^ = 0.40; Table S3).

**Table 2:** Relationship between minimum number of genera or minimum number of species and percent range area occupied during each epoch.

Variables	Eocene	Oligocene	Miocene	Pliocene	Pleistocene
Minimum genera	***p*** **<0.001**	***p*** ** = 0.004**	***p*** **<0.0001**	***p*** ** = 0.023**	***p*** ** = 0.021**
	**R^2^ = 0.73**	**R^2^ = 0.42**	**R^2^ = 0.50**	**R^2^ = 0.18**	**R^2^ = 0.16**
Minimum species	***p*** ** = 0.004**	***p*** **<0.001**	***p*** **<0.0001**	*p* = 0.057	***p*** ** = 0.042**
	**R^2^ = 0.62**	**R^2^ = 0.52**	**R^2^ = 0.49**	R^2^ = 0.13	**R^2^ = 0.12**

Significant *p*-values and subsequent R^2^ values are noted in bold. All slopes/relationships are positive.

Changes in minimum number of genera and species per family between consecutive epochs follow similar patterns to range area percent changes. Specifically, generic and species diversity on average increase from the Eocene to the Oligocene (*n* = 11, net changes of +3.3 and +5.3, respectively, although only significant at the generic level when all families are included, *p* = 0.036, Table S2). From the Oligocene to the Miocene significant gains in diversity occur at both the generic and species levels (all families with the exception of Aplodontidae increase in both generic and species diversity; n = 18, average net changes of +7.6 and +16.9, respectively; *p*≤0.01 for all comparisons, Table S2). In contrast to the lack of a clear pattern in percent range area occupied from the Miocene to the Pliocene, significant declines in generic and species diversity are observed (all families exhibit either zero change or net losses in species diversity, with the exception of Cricetidae; *n* = 25, average net changes of −6.0 and −13.1, respectively; *p*≤0.01 for all comparisons, Table S2). During the Pliocene to Pleistocene average net changes in generic and species diversity are positive, but only significantly so at the species level (*n* = 28, average net changes +0.6 and +4.7, respectively; *p*<0.01 for all species comparisons). Furthermore, there is a highly significant relationship between net changes in genera and net changes in species between consecutive epochs (*p*<0.001, R^2^≥0.56 for all comparisons) and between all consecutive epochs from the Eocene to the Pleistocene, collectively (*p*<0.0001, R^2^ = 0.79; Figure 1C, Table S4). During the Miocene to the Pliocene increases in generic diversity can and do occur with zero net changes or losses in species diversity (Felidae and Mylodontidae yield zero change in species diversity while Leporidae increases in 4 genera and declines in one species). In contrast, losses in generic diversity and zero losses or gains in species diversity occur in 18% of all families (Camelidae, Equidae, Felidae, Leporidae, and Soricidae) while 50% of all families yield zero change in generic diversity (with 6 of these 14 families also exhibiting zero change in species diversity) from the Pliocene to the Pleistocene.

### Range Size Rank Change and Concordance

**Figure 2:**
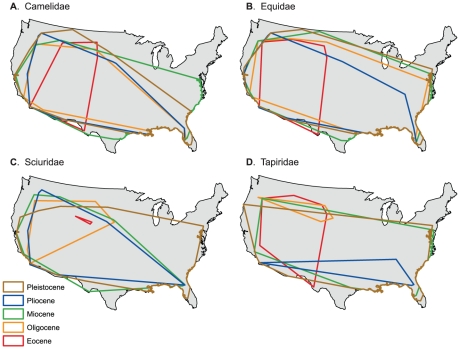
Range area polygons during each epoch for (A) Camelidae, (B) Equidae, (C) Sciuridae, and (D) Tapiridae.

Rank changes of families present across all time periods (*n* = 10) do not follow the same patterns of percent range area occupied (Table S1, Figure 2). From the Eocene to Pleistocene (Table 1, S5), Equidae held the number one ranking in all epochs except for the Pleistocene, where it was second to Cricetidae. Camelidae had the next highest ranks from the Eocene to the Pliocene, falling no lower than fourth and with a rank of 2 during the Pliocene; however, it ranked last in the Pleistocene (among taxa present since the Eocene; 20 out of 28 compared to taxa present since the Pliocene) and demonstrated the largest rank decline of all taxa. Sciuridae and Tapiridae were consistently in the bottom half of range size rankings. Sciuridae increased or maintained its rank from the Eocene through the Pliocene, falling one rank in the Pleistocene. Tapiridae alternated between decreasing and increasing in rank from the Eocene to the Pleistocene.

**Figure 3:**
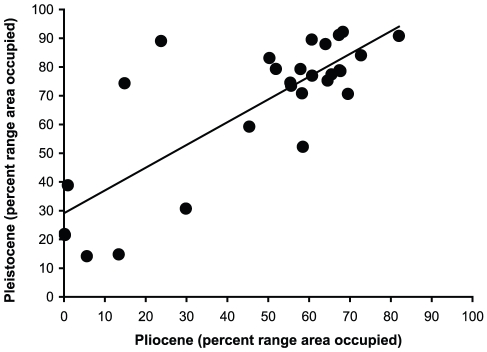
Correlation between Pliocene and Pleistocene relative range size. (R^2^ = 0.64, *p*<0.001).

Despite the majority of families either increasing or decreasing in rank at each boundary (Table 1), range size rankings within all four time intervals show statistically significant concordance (at α = .05; Table 3). Kendall's W indicates moderate concordance of ranks across older time intervals (i.e., Eocene-Pleistocene, Oligocene-Pleistocene, Miocene-Pleistocene; W>0.5; Table 3). Between the Pliocene and Pleistocene, ranks were strongly concordant (Kendall's W = 0.824), indicating constancy in relative range size, even though no family maintained precisely the same rank across the transition (Table 3). Additionally, both Pliocene range size ranks and percent range area occupied are correlated with Pleistocene range size ranks and percent range area occupied, respectively (R^2^ = 0.42, *p*<0.001; R^2^ = 0.64, *p*<0.0001, Figure 3).

**Table 3:** Concordance of ranks across geologic epochs.

Time Interval	X^2^ *	df	P value§	Kendall's W
Eocene-Pleistocene	23.400	9	**0.005**	.520
Oligocene-Pleistocene	37.169	15	**0.001**	.619
Miocene-Pleistocene	35.791	21	**0.023**	.568
Pliocene-Pleistocene	44.498	27	**0.018**	.824

* X^2^ values calculated by Friedman's test, a repeated measures comparison for k related groups.

§ Significance value (noted in bold) pertains to both the X^2^ statistic and Kendall's W, a coefficient of concordance that represents a normalization of Friedman's test.

Families present during both the Pliocene and Pleistocene were also grouped into various qualitative categories to assess taxonomic and evolutionary influences on range conservatism. At the order or higher taxonomic level (e.g., ungulates and xenarthrans), ungulates averaged 8.38 absolute rank changes (ARC), significantly greater than rodents (average ARC = 3.43; Fisher's LSD, *p* = 0.029). Two other higher level groupings of multiple families, carnivorans and xenarthrans, ranged from 4.8 to 5 mean ARC from the Pliocene to Pleistocene, but no group was statistically different from rodents, ungulates, or each other. In contrast, gross rank changes (GRC; i.e., raw relative rank changes noting negative or positive changes) per taxonomic group lack significant differences as all averaged ∼1 or less gross changes in relative rank. Furthermore, ∼50% of families within the groups carnivorans, rodents, xenarthrans, and ungulates increase/decrease in rank, with any deviations from 50% occurring in groups with an odd-number of families (e.g., increases in relative range size occur in 2 of 5 carnivorans, 3 of 7 rodents, 3 of 5 xenarthrans, and 4 of 8 ungulates).

Families containing taxa that went locally extinct in North America during the Pleistocene (*n* = 20, Table 1) have an ARC and GRC of 6.2 and −0.6, respectively. These changes are not significantly different from the ARC and GRC values of taxa containing only survivors (3.5 and 1.5; Mann-Whitney's U, *p* = 0.143, *p* = 0.476, respectively). Furthermore, there are approximately equal numbers of taxa exhibiting increases (55%) as decreases (45%) in relative range size rank in families containing victims. This is in contrast to families containing all survivors that decrease (75%) more frequently than increase (25%) in relative range size rank. Similar patterns were demonstrated when comparing families that went locally extinct in North America (ARC and GRC of 4.8 and 0.4, respectively) to those that contain at least one surviving species (ARC and GRC of 7.1 and −0.9, respectively), yielding no significant differences (Mann-Whitney's U, *P* = 0.981, *P* = 0.628, respectively). Additionally, the number of taxa increasing versus decreasing relative range size rank is approximately equal in both survivors (45% increase, 55% decrease) and victims (50% increase/decrease). Lastly, while approximately equal number of taxa increasing and decreasing in relative range size rank were found in megafauna (53% and 47%, respectively), smaller bodied taxa typically decrease in relative range size rank (66% decrease, 33% increase); however, ARC and GRC values are not significantly different (Mann-Whitney's U, *P* = 0.187; Fisher LSD, *P* = 0.690; respectively).

### Centroid

**Figure 4:**
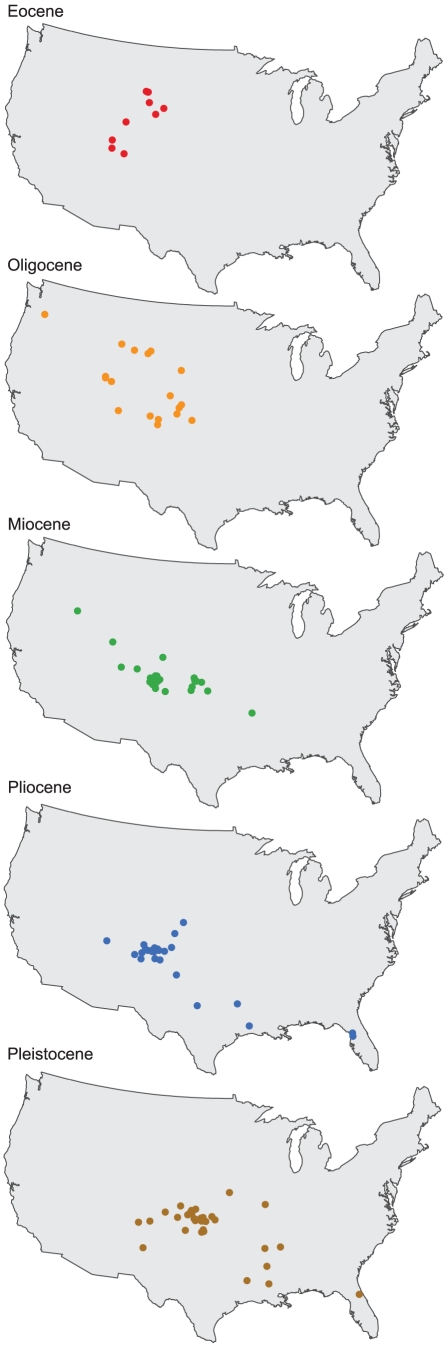
Location of centroids during each epoch analyzed.

**Figure 5:**
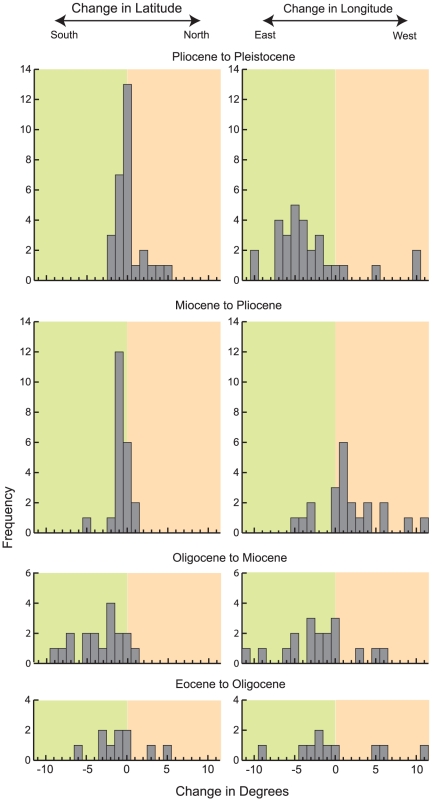
Centroid shifts between epochs, demonstrating a general southeastern trend through time.

In this study, centroids (i.e., the geometric center of the geographic range polygon) for families generally shift to the southeast from the Eocene to Pleistocene (Figures 4 and 5). The distribution of centroid shifts between epochs is more variable between the Eocene to Oligocene and Oligocene to Miocene than from the Miocene to Pliocene and Pliocene to Pleistocene (Figure 4). During the Miocene to Pliocene and Pliocene to Pleistocene, shifts in centroid latitudes are closely grouped around ±1°; however, shifts in longitude are skewed to the east (centered at ∼5°) during the Pliocene to Pleistocene and slightly skewed to the west in the Miocene to Pliocene.

## Discussion

In contrast to absolute range size which is constrained by preservation biases, percent range area occupied and range size rank standardize sampling effort, taphonomy, and continental areas across each epoch. Overall, we observe large expansions in percent range area occupied from the Oligocene to the Miocene and from the Pliocene to Pleistocene, with mixed trends leading up to the Oligocene and between the Miocene and Pliocene. Habitat availability and climactic factors may have affected ecological niches during each epoch. For example, it may be that relative range area expansion during the Miocene was a response to changing habitable niches associated with the Miocene grassland expansion [23]–[25]. The range fluctuation of Tapiridae across time (Figure 2D) is likely not only reflective of this major event, but also of forest expansions and contractions since the Eocene [26]. Furthermore, increasing relative range sizes of nearly all families from the Pliocene to the Pleistocene could be related to pronounced global cooling since the mid-Miocene climatic optimum and/or a result of glacial-interglacial cycling during the Pleistocene [27]–[28].

Alternatively, percent range area occupied may be influenced by taxonomic diversity. During any given epoch from the Eocene to the Pleistocene, the greater generic or species diversity the greater percent range area occupied, per family (Table 2). Although significant or approaching significance during the Pliocene and Pleistocene, the predictive power of taxonomic diversity is lower than during prior epochs (Table 2). Furthermore, there is only a significant positive relationship between net gains in taxonomic diversity and net gains in percent range area occupied when including all consecutive epoch comparisons from the Eocene to the Pleistocene (Figure 1, Table S3); thus, changes in taxonomic diversity at any given epoch do not correspond to proportional gains/losses at any individual epoch (with the exception of species diversity during the Eocene, Table S3). Collectively, while greater taxonomic diversity suggests greater relative range area, expansions in percent range area occupied may not necessarily be driven by changes in taxonomic diversity. Furthermore, families may be constrained by how much they can increase taxonomic diversity at any given period of time as there is a highly significant relationship between generic and species gains during each epoch and since the Eocene (Figure 1, Table S4). Although we expect a positive relationship between generic and species diversity, with species diversity exceeding generic diversity, the lack of significant deviations from this pattern (typically only occurring during the Pliocene and Pleistocene and not of significant magnitude) is surprising. Overall, adding one new genus results in the addition of ∼2.3 species (ranging from 2.0 to 3.6 from the Eocene to Pleistocene, respectively). Regardless of whether families increase or decrease in taxonomic diversity, gains/losses occur proportionally. As it is likely harder to partition resources amongst congeners than confamilials, this relationship may reflect evolutionary and ecological constraints. Additionally, if species ranges are “heritable” (e.g., [29]–[31]) and overlap more among congeners than predicted, the ability of new taxa to increase familial range area may be limited. Conversely, if range area is not heritable we might expect increased taxonomic diversity to proportionally increase relative range area. Both our family level data from the Eocene to Pleistocene and generic examples from Hadly et al. [7] from the Pleistocene to Holocene (e.g., *Canis*) lend support to the idea that range area may be heritable (as changes in range area are not significantly influenced by changes in taxonomic diversity between consecutive time periods), although neither study is explicitly designed to test this idea.

The coarseness of time bins used in this study makes it difficult to link centroid shifts to specific climatic changes as is done in Lyons et al. [20]–[21]; however, finer time bins would have prohibited the examination of rare families. The polygon for each epoch essentially represents the total range of the family for the entire epoch, with variability due to quick climatic shifts smoothed over several million years. Because of this time-averaging, centroid shifts seem to be a good representation of trends in family distributions and how these trends respond to change over longer timescales. The general southeastern shift of centroids from the Eocene through the Pleistocene (Figure 4, 5) may therefore reflect the general global cooling trend since the Eocene followed by Pleistocene glaciations [27]–[28]. Another possibility is that families are tracking particular ecosystems. Centroids for tapirs, which are good indicators of forests [26], [32], shift northeast from the Eocene to Oligocene, then southeast between the Oligocene and Pliocene, and slightly northwest in the Pleistocene. Conversely, centroids for Equidae shift southeast from the Eocene to Oligocene and remain relatively fixed in the southern Central Plains, thereafter. Although the movement of centroids southeast of the western interior since the Eocene may also be explained by potential sampling biases inherent in the North American mammal record [22], sampling biases alone do not entirely explain southeastern movement after the Oligocene as many mammalian taxa already had ranges that spanned the majority of the contiguous USA (e.g., Figure 2, S1). Instead, the southeastern movement of centroids may be a combined function of sampling, the presence of South American immigrant taxa in the southern USA in the Pliocene and Pleistocene [22], and a response to generally cooling climates since the Oligocene [27].

While range size ranks are generally conserved over deep time, individual family changes across time may be tied to major environmental transformations. For example, taxa closely tied to forest environments today (e.g., Tapiridae and Castoridae; [26], [32]–[34]) fall in relative range size rank from the Miocene to the Pliocene whereas horses maintain the top ranking from the Eocene to Pliocene (while transitioning from browsing in the Eocene to a diversity of browsing, mixed feeding, and grazing niches in the Miocene [35]). Conversely, the ecologically diverse Cricetids increase in relative range size during the Miocene to Pleistocene. These data suggest an intrinsic adaptability to ecological change within particular families. Specifically, families with greater ecological niche diversity (e.g., containing browsers and grazers, or mesic and xeric adapted taxa) may be better able to obtain larger relative range sizes than families with more specialized niches. While this may explain why camelids dramatically declined in relative range size rank (Table 1 and S5) with declining generic diversity, it is important to note that camelid diversity peaked in the Miocene [20] in North America while range size rank peaked in the Pliocene (compared to all families present since the Eocene, Table S5). Thus, there is not necessarily a direct correlation between generic or species diversity and ecological niche diversity within families. Furthermore, changes in percent range area occupied are not always matched by similar shifts in rank. For example, rodents typically have mid-to-low ranks, yet the three greatest net increases in percent range area occupied from the Eocene to Pleistocene are Cricetidae (77.2), Sciuridae (76.6) and Castoridae (73.5). This may be due to lower initial range sizes (potentially due to sampling biases), such that their potential to expand in a limited area (continental US) was greater.

When considering changes in relative range size above the family level at the Pliocene-Pleistocene boundary (Table 1), ungulates significantly shifted more ranks than rodents; however, these shifts were not accompanied by differences in the direction of change. Of the ungulates, equal numbers of taxa increased as decreased in rank size; however, the greatest rank changes noted during this interval include Elephantidae and Camelidae which increased and decreased in 17 ranks, respectively. Rodents also increased and decreased rankings, albeit at a lesser magnitude. Thus, these differences may suggest that gross body size distinctions among different mammalian orders might affect the magnitude of responses to ecological change. If the niches of larger bodied mammals show more susceptibility to short-term ecological change, then body size may serve as an intrinsic control on niche conservatism. However, families containing megafauna did not differ in relative rank changes from families that contained all taxa less than 45 kg. Thus, while ungulates observe the greatest rank changes during the Pliocene to Pleistocene this may be due to a combination of large body size and degree of dietary specialization. Furthermore, it is important to emphasize that order identity does not indicate direction of change. While rodents generally hold lower ranks, Cricetidae was ranked second in the Pleistocene. Thus, while body size correlates with an individual's home range [36], it does not necessarily correlate with family range size.

The inference of niche conservatism above the genus level by Hadly et al. [7] rests on the “constancy of relative range sizes” seen in their statistical analyses of generic and family-level ranks from the late Pleistocene to late Holocene. Our rank analysis for the Pliocene to Pleistocene interval (Table 3) indicates strongly concordant rankings from the Pliocene to Pleistocene (Kendall's W = 0.824, *p* = 0.018), a result that closely matches their concordance of rankings observed between the late Pleistocene to late Holocene transition (Kendall's W = 0.906, *p*<0.001; [7]). Thus, the significant concordance of ranks and correlations between relative range size (ranks and percent area occupied) between the Pliocene and Pleistocene further supports niche conservatism at the family level over the past ∼5 million years (Figure 3). Notably, rankings were not as strongly concordant across longer time intervals, although the influence of family identity on rank was still significant in all cases (Table 3). Perhaps niche conservatism does not operate as strongly across greater time intervals (e.g., >5 million years) and the ecological niches of closely related genera are subject to long term environmental change. These results are consistent with Peterson's [10] recent review of ecological niche conservatism, noting that niche conservatism operates over deeper timescales than previously thought but does appear to break down over time. This would suggest that the controls on niche conservatism not only vary by taxonomic level, but also timescale.

There was no significant difference in relative range size rank changes from the Pliocene to the Pleistocene between terminal Pleistocene victims and survivors or between body size categories; therefore, evidence indicating that either victims or megafauna were predisposed to extinction is lacking. These data agree with previous work that similarly demonstrates the lack of significant differences in range shifts or changes in range size between terminal Pleistocene victims and survivors from the late Pleistocene to today [20]. Although we might expect Pleistocene victims and survivors to respond differently if climate change contributed to Pleistocene extinctions [20], our data only demonstrate the lack of significant differences between these groups in range size rank changes from the Pliocene to the Pleistocene. Thus, much work remains to be done to test specific extinction hypotheses, both in North America and globally.

### Concluding Remarks

By examining the ranges of mammalian families from the Eocene through the Pleistocene, this study allows a broader view of niche conservatism which is not confounded by the appearance and disappearance of individual species over short time periods. Changes in relative range size and centroid coordinates indicate a response to environmental change at the family level. Concordance in rank, especially between the Pliocene and Pleistocene, suggests niche conservatism at the family level over longer time periods than previously demonstrated. Thus, while the location and extent of geographic ranges may vary due to environmental, climatic, and/or sampling biases, the majority of mammalian families maintain their niches relative to one another over deep time and potentially respond to environmental and/or climatic events similarly. Exceptions include ungulates that change ranks significantly more than rodents, potentially indicating that body size and diet are underlying controls of niche conservatism; thus, larger ungulates may be relatively more susceptible to environmental change. Furthermore, families containing either Pleistocene extinction victims or megafauna do not appear more prone to relative range size reductions.

Range conservation at the family level over deep time reveals the potential adaptability of a family to maintain range size dominance in the face of environmental change, if containing taxa with a moderate diversity of life history characters. For example, morphologically conservative Tapiridae fluctuates in absolute and relative range size potentially in response to the availability of forest habitat. In contrast, Equidae is able to maintain the largest relative range size from the Eocene to the Pliocene while undergoing dramatic morphological evolution [35]. While our data further suggest that ranges of higher level taxonomic classifications are less susceptible to environmental controls than individual species ranges [7], not all families are equal. Furthermore, it is important to consider both taxonomic diversity and the diversity of life history characteristics when predicting geographic ranges at higher taxonomic levels. For example, families that contain more species or more species with a greater diversity of ecological niches may prove more resilient to climatic changes then families with more specialized and/or overlapping life history variables. Therefore, deep time ecological data has the potential to provide valuable insight to understanding mammalian responses to future climate change.

## Methods

### Compilation of Geographic Range Size of Mammalian Families

We compiled a list of 35 North American mammalian families (Table 1, based on taxonomic definitions adopted by the Paleobiology Database [22]) that encompass genera that went extinct in the terminal Pleistocene [37] and genera that survived into the Holocene [7]. The spatial distribution of these families was estimated from location data downloaded from the Paleobiology Database (PaleoDB, [22]) on 20 April 2010. All occurrence points from the Eocene through the Pleistocene (55.8-0.0118 Ma) were queried. Only occurrences in the continental USA were considered to facilitate comparison with the Hadly et al. [7] dataset and because these data are well sampled and the most complete in the Paleobiology Database (compared to bordering countries). These occurrence data were then sorted into discrete time bins for the Eocene (55.8-33.9 Ma), Oligocene (33.9-23 Ma), Miocene (23-5.3 Ma), Pliocene (5.3-2.6 Ma), and Pleistocene (2.6-0.0118 Ma). The spatial extent of each family within an epoch was calculated from the minimum and maximum latitudinal and longitudinal points of the occurrence data. Rank analysis only considers families that were present in the Pleistocene and at least one other consecutive epoch (*n* = 28). In contrast, the analysis of range centroids includes all families in each epoch for which a polygon could be rendered (i.e., three or more localities per taxon, consistent with Ref. [7]).

Epoch-scale time bins allow the greatest number of families to be included in all subsequent analyses and correspond to unique periods of climate change (e.g., Oligocene cooling, Pleistocene glacial/interglacial cycling; Refs. [27]–[28]). Smaller scale designations (e.g., land-mammal ages) would have allowed for greater temporal resolution, particularly during the Miocene which is represented by periods of warming and cooling [27]; however, only the most abundant families that are present in all consecutive land-mammal ages would have been included (excluding rare and/or moderately abundant taxa and significantly reducing the number of localities per family of taxa that are present). Therefore, without resorting to only looking at orders or the most abundant families (precluding subsequent analysis of geographic range size rank over deep time due to low samples sizes), epoch-scale analyses are required.

We used ArcMap 9.3 to plot occurrence points on the contiguous United States and create range size polygons for each mammalian family. We eliminated points located in Alaska and sites that plotted outside the present shoreline. Points that were contiguous to the shoreline were included for range area calculations. Following Hadly et al. [7], separate minimum convex polygons were generated for families with the Geospatial Modeling Environment tool [38] from the coordinates of all specimens having a minimum of three points during each epoch. Completed polygons were clipped to current ocean shorelines. Political boundaries were smoothed to include the area immediately south of the US-Mexico border and northern areas of southern Ontario and Quebec between Minnesota and Maine. Range area polygons were re-projected from a geographic coordinate system (GCS North American 1983) into an equal-area projected coordinate system (USA Contiguous Albers Equal Area Conic USGS) so that areas could be calculated in ArcMap (km^2^; Table 1).

### Analysis of Geographic Range Size and Taxonomic Diversity

In addition to calculating absolute range areas per family per epoch, we calculated a percent occupied range area to control for differences in sampling. Specifically, we divided total geographic ranges by the total range area available per epoch (calculated by generating a minimum convex polygon for all mammalian families sampled per epoch, see Figure S1, Table 1, S1); a resulting percent range area occupied value was calculated for each family per epoch and statistically compared across time (Wilcoxon signed-rank test as these data were not normally distributed, as per Shapiro-Wilk tests; Table S2). The minimum number of genera and minimum number of species per family were calculated per epoch (we use the term “minimum” as only genera and species with two or more occurrences per epoch, in the Paleobiology Database [22], were included; Table S1). Minimum number of genera and species were compared over time (Wilcoxon signed-rank tests; Table S2). Linear regressions were used to assess (*i*) the relationship between taxonomic diversity (i.e., minimum number of species or minimum number of genera) and percent range area occupied per epoch (we also analyzed relationships between taxonomic diversity and range area rank; however, as results were nearly identical we only report percent range area occupied data; Table 2), and (*ii*) changes in taxonomic diversity (i.e., change in minimum number of genera or change in minimum number of species) and range area percent change, between consecutive epochs (Figure 1, Table S3).

Although our data include 15710 occurrences from 4056 unique localities (Eocene 1277, 874; Oligocene 1186, 370; Miocene 5814, 1407; Pliocene 1957, 457; Pleistocene 5476, 948; occurrences and unique localities, respectively), we cautiously examined the relationship between number of localities and percent area occupied (Figure S2, Table S1). Although there is a positive logarithmic relationship between the number of localities and percent area occupied (during each epoch and over time, Figure S2), number of localities is often unrelated to range area. For example, during the Pliocene Mammutidae has the fourth largest range area despite being represented by 19 localities. Lower range areas are also achieved regardless of the number of localities; during the Eocene ∼14% range area is occupied by Cricetidae (13 localities) and Canidae (102 localities). However, to err on the side of caution, we statistically compared changes in percent range area occupied, minimum number of genera, and minimum number of species by including all families, all families with 10 or more localities, and all families with 25 or more localities (Table S2).

### Analysis of Geographic Range Size Rank

Range areas were converted to a log_10_ scale, sorted in descending order, and assigned a relative rank in order to standardize differences in taphonomic preservation and land availability between epochs. Rank analysis was conducted across four time intervals, with varying sample sizes (Table 1): Eocene-Pleistocene, Oligocene-Pleistocene, Miocene-Pleistocene, and Pliocene-Pleistocene. Eocene to Pleistocene rank analysis only includes families for which range area could be calculated in all five epochs (Table S5). Following Hadly et al. [7], we employ non-parametric statistics (Friedman's test) to assess the constancy of ranks across each interval. Because sample size differences among the four time intervals preclude direct comparison of ranks, Kendall's W, a normalization of the Friedman test, allows us to characterize the concordance of rankings within each time interval. This essentially serves as a proxy of range conservatism at multiple timescales. Linear regressions were also used to further assess correlations between Pliocene and Pleistocene relative range size and ranks. Furthermore, we compared average relative rank changes (the absolute value, ARC) and average gross relative rank changes (an average of the net differences, GRC) of higher taxonomic groups (e.g., orders or higher), and Pleistocene families containing one or more taxon defined as megafauna (greater than or equal to 45 kg; compiled from [22], [33], [39]–[42]). We also compared families containing taxa that went extinct during the terminal Pleistocene extinction in North America with those lacking any victims [37]. Additionally, we compared families that went entirely extinct in North America with taxa containing at least one surviving taxon. The majority of statistical comparisons employ non-parametric Mann-Whitney U tests, as most data are not normally distributed; however, all statistical tests are noted and parametric tests employed when appropriate.

### Centroids

Following Lyons et al. [20]–[21], we also calculated centroids (in decimal degrees) for each polygon in ArcMap. The centroid points of family polygons represent the geometric center of each range extent. Complementing range size analysis, centroid movement provides a good average predictor of species range movement overall [20]–[21]. Here, we use centroids to demonstrate the direction and distance of family range shifts between epochs.

## Supporting Information

Figure S1 **Range area polygons for all localities included during each epoch.**
(EPS)

Figure S2 **Relationship between number of unique localities per family and percent range area occupied during each epoch.** Logarithmic trend lines for each epoch correspond to the following symbol colors: Eocene (red squares, R^2^ = 0.56), Oligocene (orange circles, R^2^ = 0.67), Miocene (green triangles, R^2^ = 0.68), Pliocene (blue Xs, R^2^ = 0.60), and Pleistocene (brown +s, R^2^ = 0.71). The black trend line corresponds to all data (R^2^ = 0.61).
(EPS)

Table S1 **Range area and rank, minimum number of genera and species, and total number of unique localities per family per epoch from the Eocene to the Pleistocene.**
(XLS)

Table S2 **Summary of Wilcoxon signed-rank tests of changes in percent range area occupied, genera, and species between consecutive epochs.**
(DOC)

Table S3 **Summary of linear regressions of net changes in minimum genera or minimum species and net changes in percent range area occupied between consecutive epochs.**
(DOC)

Table S4 **Summary of linear regressions of net changes in minimum genera and net changes in minimum species between consecutive epochs.**
(DOC)

Table S5 **Family range size ranks from the Eocene to Pleistocene.**
(DOC)
