# Supplementary material for: Mammalian Niche Conservation through Deep Time
Source: PLoS One. 2012 Apr 23;7(4):e35624. doi: 10.1371/journal.pone.0035624 (PMC3334498; doi:10.1371/journal.pone.0035624)
Supplement: Table S2 — Summary of Wilcoxon signed-rank tests of changes in percent range area occupied, genera, and species between consecutive epochs. (DOC) [file pone.0035624.s004.doc]

**Table S2. Summary of Wilcoxon signed-rank tests of changes in percent range area occupied, genera, and species between consecutive epochs.**

| Transitions | Range Area  (%) | Genera  (minimum number) | Species  (minimum genera) |
| --- | --- | --- | --- |
| Eocene - Oligocene | *p*=0.320 (+) | ***p*=0.036 (+)** | *p*=0.141 (+) |
| Eocene - Oligocene _(10)_ | *p*=0.641 (+) | *p*=0.090 (+) | *p*=0.262 (+) |
| Eocene - Oligocene _(25)_ | *p*=0.625 (+) | *p*=0.140 (+) | *p*=0.625 (+) |
| Oligocene - Miocene | ***p*<0.001 (+)** | ***p*<0.001 (+)** | ***p*<0.001 (+)** |
| Oligocene - Miocene_(10)_ | ***p*<0.001 (+)** | ***p*<0.001 (+)** | ***p*<0.001 (+)** |
| Oligocene - Miocene_(25)_ | ***p*=0.027 (+)** | ***p*=0.009 (+)** | ***p*=0.009 (+)** |
| Miocene - Pliocene | *p*=0.924 (-) | ***p*=0.001 (-)** | ***p*<0.001 (-)** |
| Miocene - Pliocene_(10)_ | *p*=0.798 (+) | ***p*=0.002 (-)** | ***p*<0.001 (-)** |
| Miocene - Pliocene_(25)_ | *p*=0.761 (-) | ***p*=0.012 (-)** | ***p*=0.005 (-)** |
| Pliocene- Pleistocene | ***p*<0.0001 (+)** | *p*=0.448 (+) | ***p*<0.001 (+)** |
| Pliocene- Pleistocene_(10)_ | ***p*<0.0001 (+)** | *p*=0.523 (+) | ***p*<0.001 (+)** |
| Pliocene- Pleistocene_(25)_ | ***p*<0.0001 (+)** | *p*=0.885 (+) | ***p*=0.004 (+)** |

*P*-values and positive/negative mean gains of more recent epochs are noted (+ or -). Analyses were performed on all families, all families represented by 10 or more localities (10), and all families represented by 25 or more localities (25). Statistically significant *p*-values are noted in bold.
