# Supplementary material for: Mammalian Niche Conservation through Deep Time
Source: PLoS One. 2012 Apr 23;7(4):e35624. doi: 10.1371/journal.pone.0035624 (PMC3334498; doi:10.1371/journal.pone.0035624)
Supplement: Table S3 — Summary of linear regressions of net changes in minimum genera or minimum species and net changes in percent range area occupied between consecutive epochs. (DOC) [file pone.0035624.s005.doc]

**Table S3. Summary of linear regressions of net changes in minimum genera or minimum species and net changes in percent range area occupied between consecutive epochs.**

| Variables | Eocene-Oligocene | Oligocene-Miocene | Miocene-Pliocene | Pliocene-Pleistocene | Eocene-Pleistocene |
| --- | --- | --- | --- | --- | --- |
| Minimum genera | *p*=0.054 | *p*=0.612 | *p*=0.107 | *p*=0.653 | ***p*<0.0001** |
|  | R^2^=0.35 | R^2^=0.02 | R^2^=0.11 | R^2^=0.01 | **R^2^=0.25** |
| Minimum species | ***p*=0.037** | *p*=0.721* | *p*=0.167 | *p*=0.786 | ***p*<0.001** |
|  | **R^2^=0.40** | R^2^=0.01* | R^2^=0.08 | R^2^<0.01 | **R^2^=0.17** |

*P*-values, and R^2^ values are noted, with statistically significant values in bold.

*Indicates negative slope/relationship, all other slopes/relationships are positive.
