# Supplementary material for: Mammalian Niche Conservation through Deep Time
Source: PLoS One. 2012 Apr 23;7(4):e35624. doi: 10.1371/journal.pone.0035624 (PMC3334498; doi:10.1371/journal.pone.0035624)
Supplement: Table S4 — Summary of linear regressions of net changes in minimum genera and net changes in minimum species between consecutive epochs. (DOC) [file pone.0035624.s006.doc]

**Table S4. Summary of linear regressions of net changes in minimum genera and net changes in minimum species between consecutive epochs.**

|  | Eocene-Oligocene | Oligocene-Miocene | Miocene-Pliocene | Pliocene-Pleistocene | Eocene-Pleistocene |
| --- | --- | --- | --- | --- | --- |
| *p-value* | ***p*<0.0001** | ***p*<0.001** | ***p*<0.0001** | ***p*<0.0001** | ***p*<0.0001** |
| R^2^ | R^2^=0.91 | R^2^=0.56 | R^2^=0.76 | R^2^=0.69 | R^2^=0.79 |
| slope | 1.98 | 2.41 | 2.39 | 3.65 | 2.32 |
| y-intercept | -1.22 | -1.42 | -1.14 | -2.5 | -0.87 |

*P*-values, R^2^ values, slopes, and y-intercepts, with statistically significant *p*-values in bold.
