# Supplementary material for: Mammalian Niche Conservation through Deep Time
Source: PLoS One. 2012 Apr 23;7(4):e35624. doi: 10.1371/journal.pone.0035624 (PMC3334498; doi:10.1371/journal.pone.0035624)
Supplement: Table S5 — Family range size ranks from the Eocene to Pleistocene. (DOC) [file pone.0035624.s007.doc]

**Table S5. Family range size ranks from the Eocene to Pleistocene.**

| Family | Eocene Rank | Oligocene Rank | Miocene Rank | Pliocene Rank | Pleistocene Rank | Average Rank | |
| --- | --- | --- | --- | --- | --- | --- | --- |
| *Equidae* | 1 | 1 | 1 | 1 | 2 | 1.2 |  |
| *Tapiridae* | 2 | 9 | 5 | 10 | 8 | 6.8 |  |
| *Camelidae* | 3 | 4 | 3 | 2 | 10 | 4.4 |  |
| *Leporidae* | 4 | 3 | 6 | 6 | 4 | 4.6 |  |
| *Canidae* | 5 | 2 | 2 | 3 | 5 | 3.4 |  |
| *Cricetidae* | 6 | 6 | 8 | 4 | 1 | 5 |  |
| *Soricidae* | 7 | 8 | 10 | 7 | 9 | 8.2 |  |
| *Ursidae* | 8 | 10 | 9 | 9 | 3 | 7.8 |  |
| *Castoridae* | 9 | 5 | 4 | 8 | 7 | 6.6 |  |
| *Sciuridae* | 10 | 7 | 7 | 5 | 6 | 7 |  |
